# Tools for App- and Web-Based Self-Testing of Cognitive Impairment: Systematic Search and Evaluation

**DOI:** 10.2196/14551

**Published:** 2020-01-17

**Authors:** Anna Pavlina Charalambous, Annie Pye, Wai Kent Yeung, Iracema Leroi, Malcolm Neil, Chryssoula Thodi, Piers Dawes

**Affiliations:** 1 School of Sciences European University Cyprus Nicosia Cyprus; 2 Division of Neuroscience and Experimental Psychology University of Manchester Manchester United Kingdom; 3 Global Brain Health Institute Trinity College Dublin Dublin Ireland; 4 University of Dundee Dundee United Kingdom; 5 Manchester Centre for Audiology and Deafness University of Manchester and the Manchester Academic Health Sciences Centre Manchester United Kingdom

**Keywords:** telemedicine, eHealth, mHealth, dementia, mild cognitive impairment, self-assessment

## Abstract

**Background:**

Tools for app- and Web-based self-testing for identification of cognitive impairment are widely available but are of uncertain quality.

**Objective:**

The objective of this study was to undertake a scoping review of app- and Web-based self-tests for cognitive impairment and determine the validity of these tests.

**Methods:**

We conducted systematic searches in electronic databases, including Google search, Google Play Store, and iPhone Operating System App Store, using the search terms “Online OR Internet-based AND Memory OR Brain OR Dementia OR mild cognitive impairment OR MCI AND Test OR Screen OR Check.”

**Results:**

We identified 3057 tools, of which 25 were included in the review. Most tools meeting the inclusion criteria assessed multiple cognitive domains. The most frequently assessed domains were memory, attention, and executive function. We then conducted an electronic survey with the developers of the tools to identify data relating to development and validation of each tool. If no response to the survey was received, Google (to identify gray literature), Google Scholar, and Medical Literature Analysis and Retrieval System Online were searched using key terms “(name of developer, if available)” AND “(the name of the tool)” to identify any additional data. Only 7 tools had any information concerning psychometric quality, and only 1 tool reported data on performance norms, reliability, validity, sensitivity, and specificity for the detection of cognitive impairment.

**Conclusions:**

The number of cognitive self-assessment electronic health tools for cognitive impairment is increasing, but most are of uncertain quality. There is a need for well-validated tools and guidance for users concerning which tools provide reliable information about possible cognitive impairment that could warrant further investigation.

## Introduction

### Background

By 2050, the number of people living with dementia is expected to increase to 152 million globally [1]. In many areas of the world, dementia remains underdiagnosed. For example, in many high-income countries, less than half of the people living with dementia receive a formal diagnosis [2]. In low- and middle-income countries (LMICs), less than 10% of the people with dementia receive a formal diagnosis [1,3-5]. Raising awareness of dementia and self-detection of cognitive decline have the potential to increase the diagnosis rate, thereby fostering appropriate support for people with dementia. Self-detection may also facilitate earlier diagnosis, a critical aspect of dementia care [6]. Several national policies relating to dementia have prioritized the need to increase the diagnosis rate and ensure a timely diagnosis of dementia [6]. Self-testing for cognitive impairment, via app- or Web-based tools, may support these aspirations by identifying people who may be developing cognitive impairment and by directing them to appropriate health and social care support services.

Electronic medical and mental health information and services (referred to as electronic health [*eHealth*]) are increasingly delivered through the internet [7]. eHealth tools include app- and Web-based tools that can screen individuals who are at risk and/or offer self-help intervention or clinical referrals for various health conditions [8]. Dramatic increases in access to and use of internet and mobile phone technology have supported the development of eHealth technology. In 1995, only 1% of the global population had an internet connection [9]. In 2019, this percentage had increased to 56.3% [10]. The majority of the worldwide population that previously did not have access to a computer or a fixed-line telephone now has mobile phones [11]. In Europe, there has been a 583% growth in internet usage between the years 2000 and 2019; over 85% of the population now has access to the internet [10].

The potential benefits of eHealth apps include improvements in health safety, improved health care efficiency and effectiveness, reduced costs, improved decision making (eg, in reaching a diagnosis), access to remote clinicians, and medical error reduction [12]. The potential benefits of eHealth apps are substantial, but there is also potential for harm. The quality, safety, and effectiveness of the majority of the proliferating eHealth apps are unknown (eg, study by Eng and Lee [13]). Health professionals have often not been involved in the development of eHealth tools, and the tools have frequently been developed without appropriate validation [14-17]. Uncertainty in the quality of eHealth tools is worrisome in relation to tools for self-identification of cognitive impairment indicative of dementia. Furthermore, if not properly validated, there is a risk of false-positive identification that may cause needless anxiety or false-negative identification that can result in a diagnosis of dementia being missed. Formal studies are urgently needed to establish the potential benefits and mitigate the harms of mobile health (mHealth) technology.

### Objectives

This review aimed to identify and assess (1) the numbers, availability, and characteristics of app- and Web-based self-assessment tools for cognitive impairment and (2) the psychometric quality of these tools to inform their future development for self-assessment of cognitive impairment.

## Methods

### Design

A systematic search was conducted between May 2017 and May 2018 by researchers at the University of Manchester, United Kingdom. We identified Web-based tools through the Google search engine and mobile phone and tablet apps through Google Play and the iPhone Operating System (iOS) App Store. The search terms we used were “Online OR Internet-based AND Memory OR Brain OR Dementia OR mild cognitive impairment OR MCI AND Test OR Screen OR Check.” We searched the iOS App Store and Google Play using the same search terms as in Google search, with the exception of the “online” and “internet-based” search terms. We screened the first 100 results we identified in each search for relevance according to the inclusion or exclusion criteria. Around 75% of *clicks* are in relation to the first 20 hits obtained [18]. We evaluated the first 100 results as a liberal criterion to capture all the research results that users would likely encounter. We completed a follow-up search in November 2018 and a further follow-up search in February 2019 to check that the tools identified in the first search were still available on the Web.

### Inclusion and Exclusion Criteria

A tool was suitable for inclusion if it (1) was designed to be a self-administered cognitive tool, (2) was hosted on the Web or as a mobile phone app, (3) was a tool intended for detection and/or assessment of (all cause) dementia and/or mild cognitive impairment (MCI), and (4) is available for free or at a low cost (≤£5). A sum of £5 was chosen as an upper limit for cost to select tools that are readily available and are within the average price range for mobile apps [19]. Games; puzzles; *brain training* apps; IQ, vocational, or academic achievement tools; tools for specific learning disability (eg, dyslexia); or tools that estimate future risk of dementia based on lifestyle factors were excluded as these tools do not measure current cognitive ability.

The first reviewer screened all titles obtained through each search, identifying candidate tests for inclusion. The second reviewer then screened 10% of the titles to ensure consensus opinion. Both reviewers held PhDs in cognitive psychology. Both reviewers evaluated all tools identified against the inclusion criteria, and in instances of disagreement, the third reviewer acted as an arbitrator and decided whether the tool met the criteria for inclusion.

### Survey of Psychometric Quality

We identified a point of contact for the owner or developer of each tool from the respective apps or websites and sent an email invitation to complete an electronic survey about the tool. The survey was adapted from a postal survey of tests or batteries for assessment of MCI [20]. We obtained permission to adapt the survey from the author of the original version. The survey contained questions about the content of the tool (ie, the cognitive domains it assesses), the duration of the test, normative data, and whether validity and reliability have been established (see Multimedia Appendix 1).

We collected the survey data over a 3-month period. In week 1, we sent out a covering email and survey link to the points of contact identified for each tool. In the subsequent 4 weeks, we sent weekly follow-up emails to those who had not responded and fortnightly reminder emails thereafter. Those who had not responded after 12 weeks were not contacted any further. For those tools for which we received no response to the survey or for which we could not identify and/or contact the owner of the tool, we conducted supplementary searches. Supplementary searches were run on Google (for gray literature), Google Scholar, and Medical Literature Analysis and Retrieval System Online (for published or peer-reviewed articles) to identify information relating to the development and validation of each tool. The search terms used were the name of developer (if available) AND the name of the tool. We screened the first 100 results, sorted by relevance. We then downloaded relevant titles and Web pages and saved the Web page links or relevant papers. Finally, we extracted data relating to the development and validation of each tool from the material we had identified in supplementary searches using a data entry table based on the same parameters of the survey questionnaire.

## Results

### Search Findings

**Figure 1:**
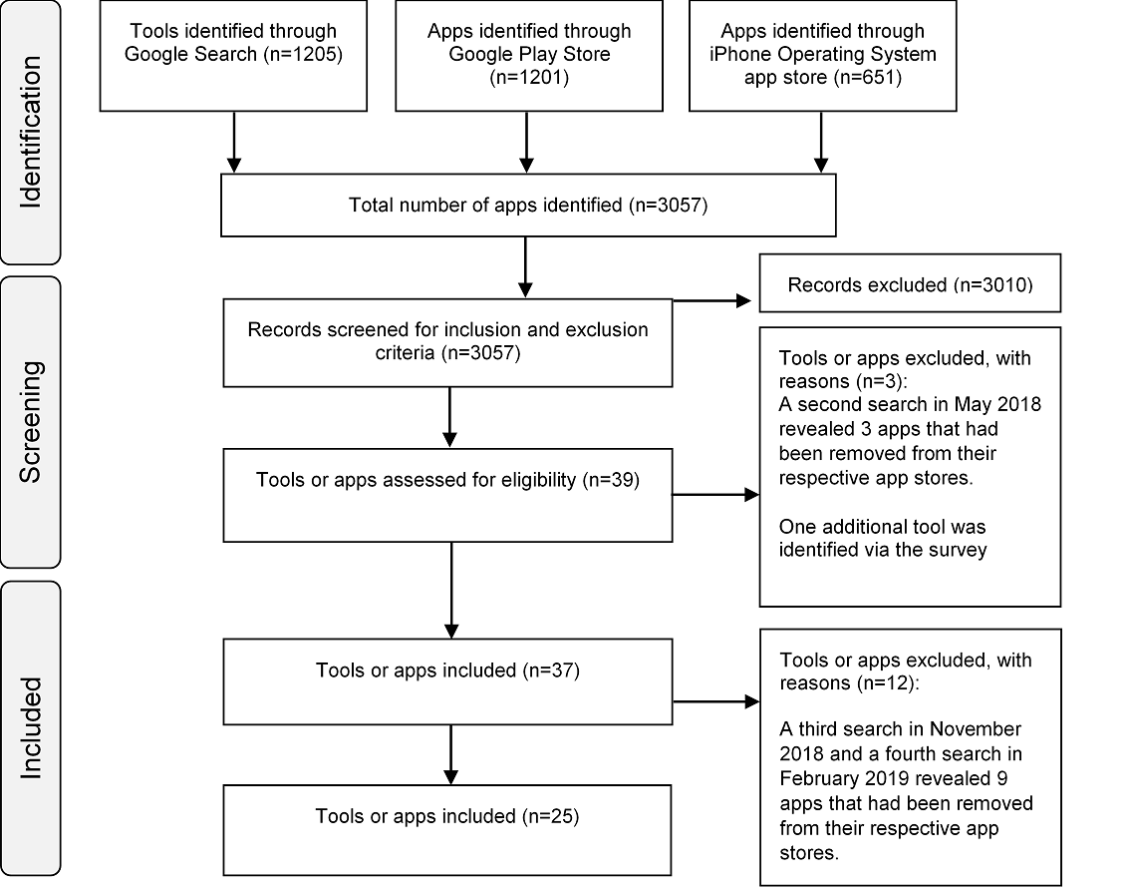
Preferred Reporting Items for Systematic Reviews and Meta-Analyses flow diagram indicating the app or tools search and screening process.

**Figure 2:**
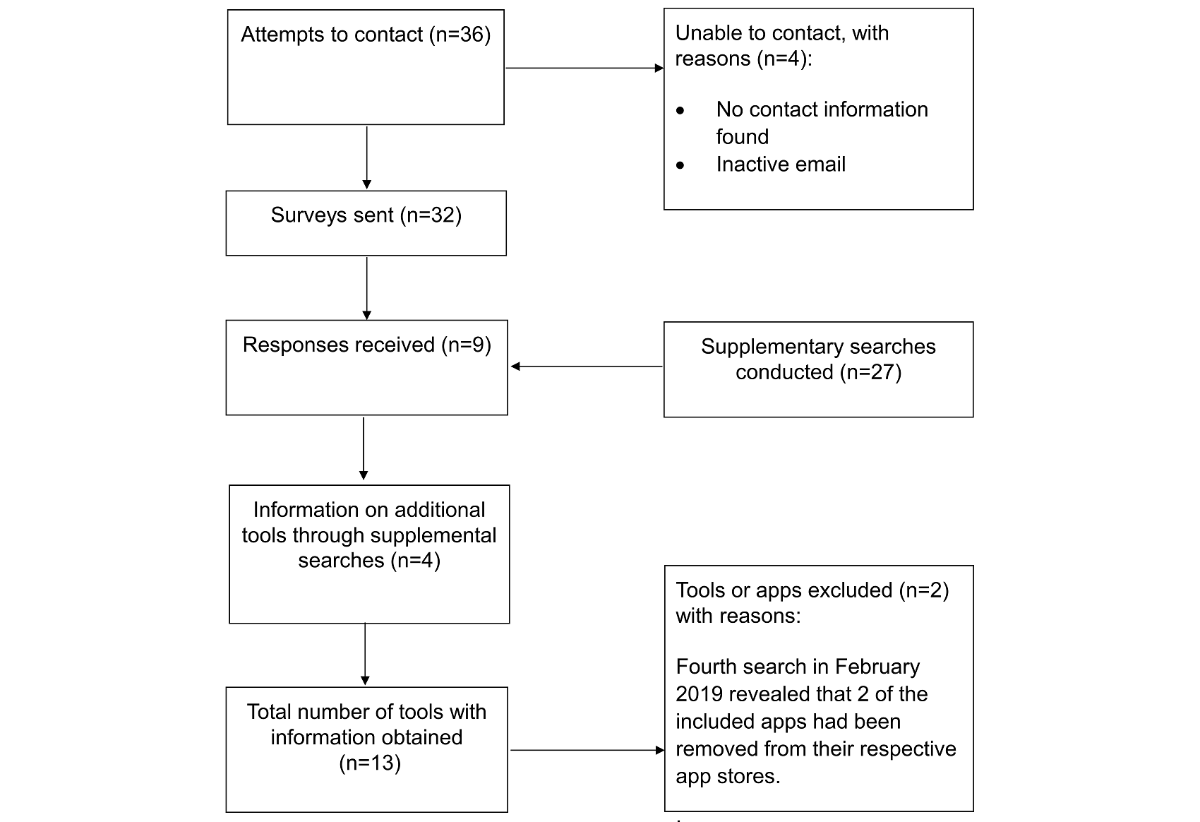
Preferred Reporting Items for Systematic Reviews and Meta-Analyses flow diagram describing the Web-based survey and supplemental searches.

We identified 3057 tools (apps and websites) after searching Google search (n=1205), Google Play (n=1201), and iOS App Store (n=651; Figure 1). The initial search identified 39 tests that fit the inclusion criteria. After a follow-up search in May 2018, we removed 3 tools after we found that they had been removed from their respective app stores, leaving us with 36 tools. We sent the survey to 32 out of the 36 tools identified. For 4 tools, we were unable to identify a contact email or emails could not be delivered, and so we were unable to contact the test developers. After the survey had been completed, 1 extra tool was added as a test developer indicated its existence, resulting in 37 tools. Follow-up searches revealed that 12 tools had been removed from their app stores, leaving 25 tools included in the review (Table 1). After sending out the online survey, we obtained responses for 9 tools. Subsequently, we obtained information for 4 additional tools through supplementary searches (see Figure 2).

**Table 1:** Cognitive domains assessed by each app- and Web-based cognitive tool.

Test name	Platform	Attention	Memory	Executive function	Visual spatial ability	Other	Not available
BrainTest (electronic Self-Administered Gerocognitive Examination)	1^a^ and 2^b^	✓^c^	✓	✓	✓	✓	—^d^
BrainCheck	1	✓	✓	✓	✓	—	—
MemTrax–The Online Memory Screening Test (free version)	1	✓	✓	—	—	✓	—
MemTrax Proprietary	1	✓	✓	✓	✓	✓	—
Self-Assessment of Cognition	1	—	✓	✓	—	—	—
Husketest	1 and 3^e^	—	✓	—	—		—
Dementia Screener	2	✓	✓	✓	—	✓	—
DANA^f^ Brain Vital	2 and 3	—	—	—	—	✓	—
DANA Modular	2 and 3	—	—	—	—	✓	—
Cogniciti	1	✓	✓	—	—	—	—
Savonix Mobile	2	✓	✓	✓	—	✓	—
Imprint Memory Assessment	1	—	✓	—	—	—	—
Memory Quiz	1	—	✓	—	—	—	—
Dementia Test	1	—	✓	—	—	—	—
RateMyMemory	1	—	✓	—	—	—	—
Daily Mail Dementia Quiz	1	—	✓	—	—	—	—
Cognitive Function Test	1	—	—	—	—	—	✓
The Cleveland Clinic Brain Check-Up	1	—	✓	—	—	—	—
Mindcrowd	1	—	✓	—	—	—	—
MyBrainTest	1	—	✓	—	—	—	—
Memory Health Check	1	—	✓	—	—	—	—
On Memory	1	—	✓	—	—	—	—
Psychology Today Memory Test	1	—	✓	—	—	—	—
Brainlab Cognition	3	—	—	—	—	—	✓
Dementia Test–Risk Calculator of Dementia	3	✓	✓	—	—	✓	—
MMSE^g^	2	—	—	—	—	—	✓
Total	—	8	21	6	2	7	3

^a^1 signifies Google search.

^b^2 signifies Google Play Store.

^c^Assessed domain.

^d^Data not assessed/data not available.

^e^3 signifies iPhone Operating System App Store.

^f^DANA: Defense Automated Neurobehavioral Assessment.

^g^MMSE is not related to the Mini–Mental State Exam [20]

We identified 17 of the 25 tools from searching Google search, 2 by searching the iOS platform, 4 by searching Google Play, 1 was identified both in Google search and Google Play, and 1 in Google Play and iOS platform (Table 1)*.* The time of test completion was reported to range from 1.5 min to 30 min. One of the Web-based cognitive tools (BrainTest [Electronic Self-Administered Gerocognitive Examination; eSAGE] tool [21]) was the digital version of the Self-Administered Gerocognitive Examination (SAGE) [22]. Most of the tools purported to assess multiple cognitive domains (Table 1). The most frequently assessed cognitive domain was memory (tested by 21 tools), then attention (8 tools), followed by executive function (6 tools). Cognitive domains that were less frequently assessed (the *Other* category) included orientation, language, fluency, and reaction time. If the cognitive domains tested by an included tool were not explicitly stated or could not be identified by reading the instructions of the tool, it was reported in Table 1 as not available.

A total of 6 survey respondents provided information concerning the collection of normative data, reliability, validity, and sensitivity/specificity for the detection of cognitive impairment (Table 2). We identified psychometric data only for 1 additional test in supplementary searches. For the rest of the tests, no psychometric data were available.

**Table 2:** Summary of availability of psychometric test data.

Test name	Normative data	Reliability	Validity	Sensitivity and specificity^a^
BrainTest (electronic Self-Administered Gerocognitive Examination) [21]	✓^b^	✓✓^c^	✓	✓✓
BrainCheck [23]	✓✓	✓✓	✓✓	✕^d^
MemTrax–The Online Memory Screening Test (free version) [24]	✕	✕	✕	✕
MemTrax Proprietary [24]	✓✓	✓	✓	✕
Self-Assessment of Cognition [25]	✓	✓	✕	✕
Husketest [26,27]	✓✓	✕	✓✓	✕
Dementia Screener [28]	✕	✕	✕	✕
DANA^e^ Brain Vital [29]	✓✓	✓✓	✓✓	✓✓
DANA Modular [30]	✓✓	✓✓	✓✓	✓✓
Cogniciti [31]	✓✓	✓✓	✓✓	✕
Savonix Mobile [32]	✕	✕	✕	✕
Imprint Memory Assessment [33]	✕	✕	✕	✕
Memory Quiz [34]	✕	✕	✕	✕
Dementia Test [35]	✕	✕	✕	✕
RateMyMemory [36]	✕	✕	✕	✕
Daily Mail Dementia Quiz [37]	✕	✕	✕	✕
Cognitive Function Test [38]	✕	✕	✕	✕
The Cleveland Clinic Brain Check-Up [39]	✕	✕	✕	✕
Mindcrowd [40]	✕	✕	✕	✕
MyBrainTest [41]	✕	✕	✕	✕
Memory Health Check [42]	✕	✕	✕	✕
On Memory [43]	✕	✕	✕	✕
Psychology Today Memory Test [44]	✕	✕	✕	✕
Brainlab Cognition [45]	✕	✕	✕	✕
Dementia Test–Risk Calculator of Dementia [46]	✕	✕	✕	✕
MMSE^f^ [47]	✕	✕	✕	✕

^a^To detect dementia or mild cognitive impairment.

^b^One tick indicates data reported to be in preparation.

^c^Two ticks indicate data currently available.

^d^A cross indicates no data available or no response.

^e^DANA: Defense Automated Neurobehavioral Assessment.

^f^MMSE is not related to the Mini–Mental State Exam [20]

### BrainTest (Electronic Self-Administered Gerocognitive Examination)

BrainTest is based on the SAGE [22], a brief cognitive assessment for identification of MCI and early dementia. Developers reported that they expected to have normative data by the end of 2019. Spearman correlations between eSAGE with SAGE (r_s_=0.88), Montreal Cognitive Assessment (MoCA; r_s_=0.76), and Mini-Mental State Examination (MMSE; r_s_=0.67) were strong, demonstrating high convergent validity. The developers found no difference between the eSAGE and SAGE with regard to sensitivity or specificity in differentiating people without dementia (MCI or normal) from those with dementia [48]. The sensitivity and specificity of eSAGE were 90% and 87%, respectively, for differentiating dementia from normal cognition and were 90% and 75%, respectively, for differentiating MCI from dementia [48]. eSAGE reportedly had 90% specificity and 71% sensitivity in differentiating those with cognitive impairment (MCI and dementia) from those with normal cognitive function [48]. The developers of BrainTest reported that data on the test-retest reliability of eSAGE were available; however, they did not share or identify any information on reliability.

### BrainCheck

BrainCheck offers a collection of neurocognitive tests intended to track cognitive health over time. According to its developers, BrainCheck has a normative database that contains more than 20,000 test results, but they did not provide any further details. BrainCheck had high 7-day test-retest reliability, with correlation coefficients ranging from 0.6 to 0.9 by subtest. The BrainCheck website reported data on sensitivity and specificity of identifying traumatic brain injury versus normal cognition [49], but no data with regard to dementia or MCI were reported. BrainCheck developers reported that a publication was underway reporting the validity of BrainCheck in relation to dementia.

### MemTrax

MemTrax is a test of recognition memory that is intended for early detection of memory problems that may be indicative of dementia. Normative data are reportedly available for the proprietary version of the MemTrax test. In a 2011 validation study, the developers gathered data from 868 individuals from 25 sites (including community events, senior citizen centers, and retirement living communities in the San Francisco Bay Area). The age range of participants was 40 to 97 years; 68.7% were female with formal education ranging from 6 to 21 years [50]. Recognition memory declined with age, and the decline was accompanied with a greater than 3-fold increase in variability over the age range. Individuals with more than 13 years of education had higher scores than those with fewer years of formal education [50]. The developers reported that test-retest reliability and convergent validity data for MemTrax (vs MoCA) [51] were being collected.

### Self-Assessment of Cognition

Self-Assessment of Cognition (SAC) is a brief cognitive screening tool designed to give older adults and their health care professionals information about memory and cognitive functioning. The developers reported that SAC has normative data from a combined sample of 206 residents of long-term care facilities for older adults (manuscripts for both studies were reportedly in preparation). The developers reported that they are collecting reliability and validity data.

### Husketest

Husketest is a multiple-choice picture recognition test. The developers of Husketest reported that they collected normative data from 795 individuals with an age range of 4 to 86 years. They also reported small effects of education and age on performance and that the test suffers from a ceiling effect, which may limit the sensitivity of the test. The developers provided no further details.

### Defense Automated Neurobehavioral Assessment Modular

This US Food and Drug Administration (FDA)–approved tool comprises a suite of 8 cognitive tests and 7 psychological surveys. The developers gathered normative data from 814 adult military veterans (71% male) aged between 18 and 64 years [52]. The test-retest reliability of Defense Automated Neurobehavioral Assessment (DANA) subtests procedural reaction time (PRT) and simple reaction time was 0.75 and 0.81, respectively [52]. The developers assessed the sensitivity of DANA in detecting MCI and Alzheimer Disease (AD) in a pilot study with 7 patient and caregiver dyads [53]. The group with AD or cognitive impairment performed worse than the caregivers for all the subtests of DANA, apart from simple reaction time [53]. Finally, the developers reported relationships among 3 DANA subtests, namely, memory search, PRT, and spatial processing with MMSE scores [54], but they did not report the correlation values.

### Cogniciti

Cogniciti is a self-assessment tool that is intended to be used by individuals to determine whether they should raise their concerns about memory with their primary care provider. Cogniciti includes (1) spatial working memory, (2) Stroop interference, (3) face-name association, and (4) letter-number alteration subtests. The developers collected normative data from 361 healthy adults aged 50 to 79 years [55]. Internal consistency (Cronbach alpha) of the face-name association test was below acceptable levels (alpha=.62), whereas consistency was excellent (alpha=.96) for the Stroop interference subtest. There were insufficient trials to calculate the internal consistency for the other 2 tasks. The developers reported retest-reliability ranging between 0.49 and 0.82 for individual subtests and 0.72 for the overall score. The developers calculated correlations between age and performance for each subtest as a measure of construct validity. These correlations were small to medium in size: −0.20 to 0.31. They examined intersubtest correlations as a measure of convergent validity, and these correlations were again small to medium in size: −0.27 to 0.30.

## Discussion

### Principal Findings

This is the first review of the quantity and quality of app- and Web-based self-assessment tools for cognitive impairment. We identified 25 tools via Google search, Google Play, and iOS platform searches, but only 7 tests had any information concerning psychometric quality, and only 1 tool (DANA) reported data on performance norms, reliability, validity, and sensitivity or specificity for the detection of cognitive impairment. The lack of information about the psychometric properties of the majority of tools indicates that although the number of cognitive self-assessment eHealth tools is increasing [11,56], their quality is unknown. The uncertain validity of the majority of tools is a concern as some tools may fail to identify people who have cognitive impairment or may cause undue anxiety by falsely identifying cognitive impairment. The focus of this review was on self-assessment tools that were not intended to inform or replace clinical decisions nor could be used to inform provision of treatment, that is, are not medical devices. However, the definition of software as a medical device is unclear, particularly in the context of software intended to identify cognitive impairment indicative of dementia. Classification of software as a medical device should consider the potential of the software to cause harm (in this case, by falsely identifying or missing true cases of cognitive impairment). We suggest that it is important to establish standards and identify ways of conveying the reliability of tools to users so that users are able to make informed choices about the tools they use and the results obtained from each tool. Developers have an ethical duty to establish the psychometric quality of the tools they offer and provide appropriate caveats on the interpretation of results obtained as well as give specific instructions for acting on the results of the self-test. eHealth apps tend to be categorized in the *Health and Fitness* or *Medical* sections of app stores. This terminology may encourage users to view these tools as legitimate sources of medical information. Formal regulation by national authorities (eg, the US FDA or the UK Medicines and Healthcare products Regulatory Agency) is a possibility, although they may struggle to keep pace with the rapid development in eHealth [57]. The FDA recently proposed that it would regulate only those apps that provide diagnostic and treatment recommendations to physicians [58,59]. This new guidance excludes all app- and Web-based self-assessment tools [60].

In the European Union’s model, developers can file an application for medical device registration with any member state of the European Union. The Conformité Européenne mark issued by the respective body in each member state is then valid throughout the European Union. The European regulatory system could offer another potential model for regulation. However, medical devices approved in Europe only need to establish performance and safety but not clinical efficacy or effectiveness [60]. The 1988 Clinical Laboratory Improvement Amendments (CLIA) model has been suggested as a possible solution for the regulation of mHealth, including mobile computing, medical sensor, and communication technologies for health care apps [8,61]. CLIA is a system for ensuring that diagnostic testing laboratories comply with US regulatory standards. Nonprofit accrediting agencies with authority to issue certification under federal CLIA standards ensure consistency of record keeping and staff training [61,62]. Larson [60] argued that a CLIA model could ensure that mHealth apps comply with basic standards, including (1) accessibility: clear language, usability, and affordability; (2) privacy and security, including data sharing with third parties and; (3) content: apps developed with health care experts contain accurate information, limit advertising, and explain monetization (eg, referral generation or sales) and conflicts of interest [17]. Existence of a recognized standard of quality for mHealth apps could provide an additional incentive for developers to establish the psychometric quality of the tools they provide. Establishing the psychometric quality of assessments requires significant investment. Developing and establishing the psychometric properties of a cognitive test requires psychometric expertise and carefully controlled testing of large numbers of people with and without cognitive impairment. If an app is made available for free or at a low cost, developers would have to either (1) have a business model that funds development and running costs without directly transferring those costs to the end user (eg, apps that make referrals to for-profit health care providers) or (2) be well funded by charity, social enterprise, or government organizations (eg, DANA developed by the US military). Development cost limitations are likely to limit the number of good quality self-assessment apps.

The tools in this review were all either free or low cost and readily accessible. eHealth tools may have the potential to address the underidentification of dementia by increasing the awareness of cognitive impairment and directing people who may have cognitive difficulties to appropriate clinical diagnostic and support services. Overall, 2 tools mentioned in this review were linked to clinical services (Cogniciti) or provided specific recommendations to speak with a doctor (eSAGE). Furthermore, 3 tests provided advice on healthy lifestyles to promote cognitive health (Daily Mail Dementia Quiz, Memory Health Check, MyBrainTest, and On Memory). However, 19 out of the 25 tools included in the review did not directly link users with a clinician or support service. The lack of clear advice or direction on what steps should be taken in the event of a failed screening is of concern; some tests may cause anxiety by identifying a possible impairment without providing advice about how to seek help. Lack of clear direction may also mean that few people may act on the results of a failed cognitive screening. We did not identify any study that evaluated the proportion of people who failed the screening or who went on to seek help. In addition, none of the studies identified barriers to acting on the results of failed tests nor investigated how help might best be provided. The lack of follow-up actions may be a serious shortcoming of most Web-based cognitive self-assessments.

Further efforts should be made to link the results of Web-based cognitive self-assessments directly to clinical services to minimize anxiety caused by identification of a possible impairment and facilitate action on the result of a failed screening. eHealth cognitive self-assessment tools could potentially utilize the growing acceptance and use of video conferencing in geriatric psychiatry care by clinicians and patients. Videoconferencing is well received by patients and clinicians [63] and may facilitate the reach of clinical services in underserved areas [64]. Video consultations may help increase diagnostic coverage particularly in LMICs. As an alternative to referral to clinical services, Web-based cognitive assessments could direct test takers to interventions delivered via the internet to support cognitive function. For example, the Imprint Memory Assessment eHealth tool links to a Web-based memory health program based on diet, exercise, cognitive training, and vascular risk monitoring [65]. Formal evaluation of the risks and benefits of a cognitive eHealth paradigm is an urgent priority.

### Conclusions

There is a need to establish the quality of cognitive self-assessment tools while maintaining their low cost and easy accessibility. A regulatory model should ensure standards of accessibility, privacy, and content. The results of app- and Web-based cognitive self-assessments should be linked to appropriate clinical and support services.

## Appendix

Multimedia Appendix 1 Survey questions.

## Abbreviations

AD: Alzheimer Disease
CLIA: Clinical Laboratory Improvement Amendments
DANA: Defense Automated Neurobehavioral Assessment
eHealth: electronic health
eSAGE: Electronic Self-Administered Gerocognitive Exam
FDA: Food and Drug Administration
iOS: iPhone Operating System
LMIC: low- and middle-income country
MCI: mild cognitive impairment
mHealth: mobile health
MoCA: Montreal Cognitive Assessment
MMSE: Mini-Mental State Examination
PRT: procedural reaction time
SAC: Self-Assessment of Cognition
SAGE: Self-Administered Gerocognitive Examination
