# Supplementary material for: Tools for App- and Web-Based Self-Testing of Cognitive Impairment: Systematic Search and Evaluation
Source: J Med Internet Res. 2020 Jan 17;22(1):e14551. doi: 10.2196/14551 (PMC6996724; doi:10.2196/14551)
Supplement: Multimedia Appendix 1 [file jmir_v22i1e14551_app1.doc]

**Appendix A.** Survey questions

| **Section** | **Topic** | **Question(s)** | |
| --- | --- | --- | --- |
| **Section 1** | General description of measure and measure development | Please enter a link or url for your tool. |  |
|  |  | Name of the tool |  |
|  |  | Is your test available | Online |
|  |  |  | On an Android device |
|  |  |  | On an Apple device |
|  |  |  | Other (Please specify) |
|  |  | Please provide a brief description of your test |  |
|  |  | What cognitive domains does your test assess? | Memory |
|  |  |  | Attention |
|  |  |  | Executive Function |
|  |  |  | Fluency |
|  |  |  | Language |
|  |  |  | Visuospatial ability |
|  |  |  | Other (please specify) |
|  |  |  |  |
|  |  |  |  |
|  |  |  |  |
|  |  | Does your test include assessment of ‘red flag’ symptoms that could indicate serious visual pathology? | Yes |
|  |  |  | No |
|  |  |  | If yes, please provide details |
|  |  | Is your test in the public domain/proprietary? |  |
|  |  | If proprietary, please describe how to obtain access to the instrument. |  |
| **Section 2** | Normative data | Have normative data been collected for your test? | Yes |
|  |  |  | No |
|  |  |  | If yes, please provide details of normative data (populations, relevant publications, tables, documents) |
| **Section 3** | Reliability, validity and responsiveness | Which, if any, of the following validity, reliability and responsiveness analyses have been undertaken with your test? | Sensitivity for detecting change over time |
|  |  |  | Convergence with similar tests |
|  |  |  | Convergence with tests of functional ability |
|  |  |  | Predictive validity |
|  |  |  | Sensitivity and/or specificity |
|  |  |  | Construct and face validity |
|  |  |  | Test-retest reliability |
|  |  |  | Internal consistency |
|  |  | Please provide details of any analyses and relevant publications. |  |
|  |  | Please describe the kind of feedback that the user receives upon completing the measure. |  |
| **Section 4** | Data collection and storage | Can your test be completed by lay people without support from trained technicians or clinicians? | Yes |
|  |  |  | No |
|  |  | What technology and hardware requirements are there for running your test? |  |
|  |  | How long does it take to complete your test in minutes? |  |
|  |  | How are test results displayed, stored and transmitted? |  |
|  |  | Are test results accessible in a real-time (or close to real-time manner? | Yes |
|  |  |  | No |
|  |  | Does your data collection and storage system allow for the provision of real-time alerts regarding significant vision change, for use by the user? | Yes |
|  |  |  | No |
|  |  | If yes, who would receive that alert? | The User |
|  |  |  | Caregivers or family members |
|  |  |  | The supervising clinician |
|  |  |  | Other (please specify) |
| **Section 5** | Quality assurance and approvals | Does your technology allow for any automation of quality assurance/ quality checking of data inputted by the user? | Yes |
|  |  |  | No |
|  |  |  | If yes, please describe |
|  |  | Has your test been accepted by any health regulatory authorities (e.g. national regulatory authority, European Medicines Agency (EMA), US Food and Drug Administration (FDA)) as a valid measure for any particular disease entity? | Yes |
|  |  |  | No |
|  |  |  | If yes, please describe. |
| **Section 6** | Availability and accessibility | Are there any physical, cognitive or psychological restrictions that might limit test completion? |  |
|  |  | Are there any culturally or linguistically adapted test versions? | Yes |
|  |  |  | No |
|  |  |  | If yes, please describe. |
| **Section 7** | Other information | If you have any other comments about your tool that we did not cover in this survey, please list them below. |  |
|  |  | Please provide an email address that we might use to contact you should we have further questions regarding your tool. |  |
